# Neural Computations Underlying Phenomenal Consciousness: A Higher Order Syntactic Thought Theory

**DOI:** 10.3389/fpsyg.2020.00655

**Published:** 2020-04-07

**Authors:** Edmund T. Rolls

**Affiliations:** ^1^Oxford Centre for Computational Neuroscience, Oxford, United Kingdom; ^2^Department of Computer Science, University of Warwick, Coventry, United Kingdom; ^3^Institute of Science and Technology for Brain-Inspired Intelligence, Fudan University, Shanghai, China

**Keywords:** consciousness, higher order thought, levels of explanation, backward masking, syntax, global workspace, attention, inattentional blindness

## Abstract

Problems are raised with the global workspace hypothesis of consciousness, for example about exactly how global the workspace needs to be for consciousness to suddenly be present. Problems are also raised with Carruthers’s (2019) version that excludes conceptual (categorical or discrete) representations, and in which phenomenal consciousness can be reduced to physical processes, with instead a different levels of explanation approach to the relation between the brain and the mind advocated. A different theory of phenomenal consciousness is described, in which there is a particular computational system involved in which Higher Order Syntactic Thoughts are used to perform credit assignment on first order thoughts of multiple step plans to correct them by manipulating symbols in a syntactic type of working memory. This provides a good evolutionary reason for the evolution of this kind of computational module, with which, it is proposed, phenomenal consciousness is associated. Some advantages of this HOST approach to phenomenal consciousness are then described with reference not only to the global workspace approach, but also to Higher Order Thought (HOT) theories. It is hypothesized that the HOST system which requires the ability to manipulate first order symbols in working memory might utilize parts of the prefrontal cortex implicated in working memory, and especially the left inferior frontal gyrus, which is involved in language and probably syntactical processing. Overall, the approach advocated is to identify the computations that are linked to consciousness, and to analyze the neural bases of those computations.

## Introduction

A theory of phenomenal consciousness has recently been described that holds that this arises when there is a global workspace with non-conceptual content (Carruthers, 2019). Non-conceptual content refers to content that is about continuous representations, for example about exactly how large an apple is, or just how red it is. The theory holds that phenomenal consciousness can be reduced to a natural (physical or physically realized) property. Further, it is held that phenomenal consciousness can be associated with first order thoughts, and does not require higher order thoughts (HOTs) (thoughts about thoughts, including reflection). Carruthers (2019) can identify no useful function performed by phenomenal consciousness, and does not take a computational approach.

As a computational neuroscientist (Rolls, 2016), I find that it is helpful in understanding brain and cognitive function to identify the computations being performed by particular brain areas to perform their functions. I therefore take here the computational neuroscience approach, to identify what may be weaknesses in and alternatives to the hypotheses described above (Carruthers, 2019), and then describe and develop further, a different, higher order syntactic thought (HOST), theory of phenomenal consciousness (Rolls, 2016). Phenomenal consciousness refers to subjective feelings, about what it feels like to have a perception, or an emotion, etc.

The first part of this paper includes a neuroscience assessment of some of the key issues in the area of neuroscience around which Carruthers builds his theory. It shows why I think that alternatives to his approach, and to global workspace theories in general (Dehaene, 2014), should be sought. This includes a new consideration of levels of explanation in neuroscience, and how causality may be assessed in relation to brain vs. mental representations. An implication is that phenomena at one level, such as phenomenal consciousness, cannot be reduced to what co-occurs at a lower level, such as physical processes. The second part of this paper outlines and develops further a higher order syntactic thought (HOST) theory of consciousness, which specifically identifies a kind of computation performed by the brain that the theory holds is present when phenomenal consciousness is reported. That goes beyond global workspace theory, which specifies a lot of processing, but not the kind that is important for consciousness. My HOST theory also identifies important functions performed by those computations which go beyond what is described in HOT approaches to consciousness.

Phenomenal consciousness refers to subjective feelings of awareness, and the main criterion in humans for establishing that it is present is reportability (Block, 2005; Rosenthal, 2005; Carruthers, 2019).

## The Global Workspace Hypothesis of Phenomenal Consciousness

It has been proposed that when information processing in the brain becomes sufficiently widespread and involves sufficient brain regions, then phenomenal consciousness occurs (Baars, 1988). Many studies have been performed that provide general support for the approach, by showing that when phenomenal consciousness is reported, cerebral processing is more extensive, and often includes activity in the prefrontal cortex (Dehaene et al., 2006, 2014, 2017; Kouider and Dehaene, 2007; Dehaene, 2014). Carruthers (2019) adheres to a global workspace approach to phenomenal consciousness.

I propose that a major weakness of the global workspace hypothesis is that it just proposes that when a certain amount of global processing is reached, phenomenal consciousness arises. That is, it does not identify the particular *kind* of neural processing that is related to consciousness. In these circumstances, the slope is very slippery. Exactly how much more global must the processing be for it to mediate phenomenal consciousness? If there is no yardstick that defines when enough global processing is occurring for phenomenal consciousness to be present, how can we use this to identify exactly what neural processing is related to phenomenal consciousness?

To give an example, some people report being able to do considerable processing, which seems to be rather global, without reporting phenomenal consciousness and it feeling like something, for example driving a car, for a short distance, which must include extensive visual and motor processing. The report may be that the person was conscious about something entirely different, such as an ongoing discussion about the nature of consciousness. Many examples that much can be performed unconsciously, that is subliminally, are described by Dehaene (2014) and in Section “Multiple Routes to Action.” Another example shows further that even when language is being used, that is not a sufficiently global level of processing for phenomenal consciousness to be present. The example is from Larry Weiskrantz, who investigated blindsight so interestingly (Weiskrantz, 1986, 1997, 1998), who used to say to me every morning in the Department of Experimental Psychology at the University of Oxford “Edmund, how are you today,” and I would reply “Fine, Larry.” Larry would then say: “Edmund, you have no right to say that. You have not reflected on it.” Weiskrantz’s point was that there is a *kind* (a type) of processing that may be taking place when we are phenomenally conscious, and that even being able to use language may not be a sufficient criterion in terms of the level and amount of processing for phenomenal consciousness to be present. I agree with Weiskrantz on this, and that is why I find the global workspace hypothesis of phenomenal consciousness to be too imprecise, with the amount of global processing required not being defined in terms of any category, but instead somewhere on a sliding scale. Weiskrantz’s views about consciousness were that reflection, which is a *kind* of information processing, is involved in consciousness. My own view, set out below, is that it is a particular kind of processing that underlies phenomenal consciousness, which is more precisely defined than reflection but which is consistent with reflection, namely the computations involved in higher order syntactic thoughts (HOSTS).

Dehaene (2014) has a global workspace space theory of consciousness that does not make as many claims about it containing only non-conceptual content etc. as Carruthers (2019). However, Dehaene’s approach deals mainly with access consciousness (for example responding to stimuli, see Block, 2005), rather than very specifically addressing phenomenal consciousness. Also, the exact computations that are involved in (mainly access) consciousness are not make explicit beyond something to do with long-range communication in the brain (Dehaene et al., 2014). Dehaene (2014) writes (p 168): “I believe that consciousness reduces to what the workspace does: it makes relevant information globally accessible and flexibly broadcasts it to a variety of brain systems.” Dehaene’s approach is though helpful in identifying some brain areas that seem to be important in (mainly access) consciousness, including spread to the global workspace to include areas such as the prefrontal cortex, ventromedial prefrontal cortex, area 10 of the anterior prefrontal cortex, posterior cingulate cortex, and precuneus. Elsewhere (p. 108) Dehaene (2014) states that reasoning may involve consciousness: “rationally thinking about a problem.” That is starting to become close to HOST theory, in that at least Dehaene has in mind something to do with serial thinking of the type that might be implemented by a system capable of syntax, which might be a set of first order thoughts attempting to solve a problem. Further, he believes that neurons with large dendrites in the prefrontal cortex may be useful for the global workspace with the long-range connectivity needed, and even relates this to language. He writes (p 173) that FoxP2, a gene specific to humans, modulates our language networks, and generates very large dendrites. Dehaene (2014) may be thinking beyond a global workspace as being important in consciousness, toward the kind of computation that brain systems implement when consciousness is present. Addressing the type of computation that is involved is a key approach taken in HOST theory [see section “A Higher Order Syntactic Thought (HOST) Theory of Phenomenal Consciousness”].

Shea and Frith (2019), like me, believe that global workspace theories are inadequate, and that some type of metacognition may be involved. In my Host theory [see section “A Higher Order Syntactic Thought (HOST) Theory of Phenomenal Consciousness”], I propose a particular type of metacognition that is involved in phenomenal consciousness, and what the advantages are of that type of metacognition.

For these reasons, I hold that current global workspace theories are inadequate theories of phenomenal consciousness (Rolls, 2011, 2012b, 2016), though they do help to make progress with understanding at least access consciousness.

## Attention and Phenomenal Consciousness

Carruthers (2019) also considers what we learn about consciousness from attentional phenomena. Apparently contrary to global workspace hypotheses is research on attention and phenomenal consciousness. For example, inattentional blindness indicates that we only have phenomenal consciousness for visual stimuli to which we are selectively paying attention (Mack and Rock, 1998; Simons and Rensink, 2005), which is when the workspace is not global, but focused by attention. For example, if we pay attention to a baseball game, and are told to watch the ball, we are not aware of a gorilla wandering boldly across the scene (Simons and Chabris, 1999). This can be accounted for by the well-understood mechanisms of attention. In a complex spatial scene, the receptive fields of inferior temporal cortex neurons decrease from approximately 70 degrees in diameter to a few degrees, about the size of a typical object (Rolls et al., 2003; Aggelopoulos and Rolls, 2005). This can be accounted for by local lateral inhibition decreasing the size of inferior temporal cortex Gaussian receptive field profile neurons (Trappenberg et al., 2002). That would mean that if fixating the ball in the ball game, the inferior temporal cortex neurons would not respond to the gorilla, who was not being fixated and was several degrees from the fovea.

Top down attention can again influence what enters phenomenal consciousness, but this is not surprising, for top–down attentional mechanisms facilitate the representations of objects to which we are paying attention by a top–down bias, relative to other objects (Rolls and Deco, 2002; Deco and Rolls, 2004, 2005a,2005b; Rolls, 2016).

## Levels of Explanation in Neuroscience, and the Implication That Phenomena at One Level, Such as Phenomenal Consciousness, Cannot Be Reduced to What Co-Occurs at a Lower Level, Such as Physical Processes

We can now understand brain processing from the level of ion channels in neurons, through neuronal biophysics, to neuronal firing, through the computations performed by populations of neurons, and how their activity is reflected by functional neuroimaging, to behavioral and cognitive effects (Rolls, 2016). Activity at any one level can be used to understand activity at the next. This raises the philosophical issue of how we should consider causality with these different levels (Rolls, 2016). Does the brain cause effects in the mind, or do events at the mental, mind, level influence brain activity?

What is the relation between the mind and the brain? This is the mind-brain or mind-body problem. Do mental, mind, events cause brain events? Do brain events cause mental effects? What can we learn from the relation between software and hardware in a computer about mind-brain interactions and how causality operates? Neuroscience shows that there is a close relation between mind and matter (captured by the following inverted saying: ‘Never matter, no mind’).

Carruthers (2019) holds that phenomenal consciousness can be reduced to a natural (physical or physically realized) property. This, for him, takes much of the mystery out of phenomenal consciousness, for it is just a matter of matter, and simplifies his approach to all the questions raised by phenomenal consciousness. But is it reasonable to argue that one can reduce what is at a very high level in the processing system to the physical properties that implement the processing at a lower level? I do not think so. To make this point, we need to consider how different levels of the system, such as the neuronal level and the computational function being performed, relate to each other. This is part of the very big problem of the relation between the mind and the brain. Here is my approach to this.

One possible view that has been described (Rolls, 2016) is that the relationship between mental events and neurophysiological events is similar to the relationship between the program running in a computer and the hardware of the computer. Does the program (the software loaded onto the computer usually written in a high-level language such as C or Matlab) ‘cause’ the logic gates (TTL, transistor-transistor logic) of the hardware to move to the next state? And does this hardware state change ‘cause’ the program to move to its next step or state? Those interested in the philosophy of the ‘mind-brain’ problem need to provide a clear view on this computational issue in computers, as that is a well-formulated problem.

I propose that one way to think about this is that when we are looking at different levels of what is overall the operation of a system, causality can usefully be understood as operating within levels (causing one step of the program to move to the next; or the neurons to move from one state to another), but not between levels (e.g., software to hardware and vice versa). That is, if the events at the different levels of explanation are occurring simultaneously, without a time delay, then my view is that we should not think of causality as operating between levels, but just that what happens at a higher level may be an emergent property of what happens at a lower level. This is the solution I propose to this aspect of the mind-brain problem.

Following this thinking, when one step of a process at one level of explanation moves to the next step in time, we can speak of causality that would meet the criteria for Granger causality where one time series, including the time series being considered, can be used to predict the next step in time (Granger, 1969; Bressler and Seth, 2011; Ge et al., 2012). In contrast, when we consider the relationship between processes described at different levels of explanation, such as the relation between a step in the hardware in a computer and a step in the software, then these processes may occur simultaneously, and be inextricably linked with each other, and just be different ways of describing the same process, so that temporal (Granger) causality does not apply to this relation between levels, but only within levels. The whole processing can now be specified from the mechanistic level of neuronal firings, etc. up through the computational level to the cognitive and behavioral level.

Sometimes the cognitive effects seem remarkable, for example the recall of a whole memory from a part of it, and we describe this as an ‘emergent property,’ but once understood from the mechanistic level upwards, the functions implemented are elegant and wonderful, but understandable and not magical or poorly understood (Rolls, 2016). We can say here that the way in which a discrete attractor network settles by its collective computation into a low energy basin of attraction to solve a computational problem is different *in kind* from a set of individual neurons firing action potentials, or from the transmitter being released onto each of the 10,000 synapses in the typically 100,000 neurons in the cortical attractor network (Rolls, 2016). In this sense, I do not think that a claim that all the properties of the system, including its emergent properties, can be reduced to what might be happening in noisy ion channels in synapses. Of course, what is happening simultaneously at different levels of the system is essential for its operation. But what is happening at each level may be thought of as a different kind. Computing invariant representations of objects, or recalling memories from a small retrieval cue, can be thought of as different kinds of operation (Rolls, 2016). In this situation, I am not persuaded by Carruthers’s (2019) claim that all the properties of the system at all levels can be reduced to operations at a low level of physical processing. There appear to be differences in the kinds of processing at different levels.

Now I argue below for my HOST theory of phenomenal consciousness that at a very high level of the system, and only in a particular part performing a particular kind of computation, namely HOST processing, the top level of this system, another emergent property may be phenomenal consciousness. It just happens to be a property of this particular system, when it is doing the kind of computation that I will describe, that it feels like something. So I will argue that what is computed at high levels of the system can be different in kind, and have different properties, to what may be happening at a very low level of the system, such as ion channels at synapses, or neuronal action potentials. I therefore believe there is something different in kind about phenomenal consciousness from neuronal firing or synaptic transmitter release, and that makes my theory of phenomenal consciousness very different to the theory of Carruthers (2019) who reduced phenomenal consciousness to the level of physical properties of the components that implement the computations.

Another point of importance is whether we can consider that phenomenal consciousness has a function, that is, is causal. Carruthers (2019) thinks that phenomenal consciousness is causal, because we can detect it, by reporting whether at a particular time we are phenomenally conscious of a particular event. My view, which follows from my levels of explanation approach just described, is that causality can best be considered to operate within a level, rather than between levels. And I argue that the HOST computations to which consciousness is related do have very useful functions, which implies causal effects, as I will describe. So I may wish to say the phenomenal consciousness may have causal effects within its own level of processing; but that what matters more practically is that the HOST-related computations themselves have very useful functions in solving credit assignment problems, and do this by operating within that computational level, not at the level of subjective experience.

## Phenomenal Consciousness Can Include Conceptual as Well as Non-Conceptual Content

In neuroscience, a fundamental distinction is made between categorical or discrete, and continuous, representations (Rolls, 2016). Major examples of continuous representations are spatial representations, which are inherently continuous. These are frequently modeled by neurons with Gaussian receptive fields that overlap partly with each other to cover the whole of the space, and form a continuous attractor network (Rolls, 2016). I agree with Carruthers (2019) that continuous representations, of for example space, the magnitude of a sensory or reward-related input, the exact color, etc. must be represented in phenomenal consciousness.

However, I am puzzled by Carruthers’ (2019) failure to include conceptual representations in phenomenal consciousness. Conceptual representations are representations where there are distinct categories, such as a banana vs. an apple, a table vs. a chair, or the face of one person vs. the face of another person. Another example is provided by nouns, which frequently refer to categories of object, such as the word ‘banana,’ or the word ‘apple.’ In neuroscience, we refer to these as discrete representations. My view is that these discrete representations can also be included in phenomenal consciousness. Take for example my mental arithmetic about numbers, which are discrete representations. If I perform mental arithmetic, performing some manipulation of the numbers in a list such as placing them into reverse order, or multiplying each number by 2 to the power of its position in the list, being told that I have made an error somewhere, and then thinking about what I did before, and trying to correct it, then that is exactly the type of computation about discrete representations that is represented in at least my phenomenal consciousness. My HOST theory of consciousness applies to conceptual as well as for non-conceptual representations, both of which can benefit from the HOST type of computation, as described below.

## Phenomenal Consciousness Is a Matter of ‘Kind’

Carruthers argues that phenomenal consciousness is ‘all-or-none,’ either present, or not. I tend to agree with him. But that seems somewhat inconsistent with a global workspace hypothesis, in which when some sort of sliding threshold of a continuous variable ‘globalness’ has been reached, consciousness is suddenly present in an all or none way. My HOST theory proposes that the unitary property of consciousness is related to its implementation by a limited capacity syntactic processor that can only handle one stream of processing at a time. The computational system thus performs a particular kind of processing.

## A Higher Order Syntactic Thought (Host) Theory of Phenomenal Consciousness

This Section updates and summarizes my HOST theory of consciousness ([citeskum]BR73,BR78,BR79,BR80,BR82,BR85[citeekum]Rolls, 1997b, 2008, 2010, 2011, 2012b, 2016).

### Multiple Routes to Action

A starting point is that many actions can be performed relatively automatically, without apparent conscious intervention. An example given above is driving a car for a short time if we are thinking about something else. Another example is from the areas of subliminal processing, for example the identification of a visual stimulus that can occur without conscious awareness, as shown by backward masking experiments (Rolls and Tovee, 1994; Rolls et al., 1994b, 1999; Rolls, 2003). In these psychophysical and neurophysiological studies, it was found that face stimuli presented for 16 ms and followed immediately by a masking stimulus were not consciously perceived by humans, yet produced above chance identification, and firing of inferior temporal cortex neurons in macaques for approximately 30 ms. If the mask was delayed for 20 ms, the neurons fired for approximately 50 ms, and the test face stimuli were more likely to be perceived consciously (Rolls, 2003). This provides evidence that conscious processing may have a higher threshold in sensory processing than implicit processing that can lead to behavioral actions (such as identifying the stimulus). Consistent with this neurophysiology and the comparison with humans, neurons in the human temporal lobe cortex also have larger responses to stimuli on trials on which the human was aware of having seen the stimulus (Quiroga et al., 2008). Further examples of larger neural responses when stimuli are perceived are described by Dehaene (2014).

It is suggested that part of the adaptive value of a higher threshold for conscious awareness is that if conscious processing is inherently serial and slow (because it may involve syntactic operations in ways that we may be starting to understand (Rolls and Deco, 2015), it may be maladaptive to interrupt it unless there is a high probability that the interrupting signal does not arise from noise in the system. Part in fact of my HOST theory of consciousness (see Section “A Computational Hypothesis of Phenomenal Consciousness” onwards) is that it provides a computational reason why the threshold for information to reach consciousness is higher than the threshold for information to influence behavior in what is referred to as subliminal processing (Dehaene et al., 2006).

Another example is much of the sensory processing and actions that involve the dorsal stream of visual processing to the parietal cortex, such as posting a letter through a letter box at the correct orientation even when one may not be aware of what the object is (Milner and Goodale, 1995; Milner, 2008; Goodale, 2014). Another example is blindsight, in which humans with damage to the visual cortex may be able to point to objects even when they are not aware of seeing an object (Weiskrantz, 1997, 1998, 2009). Similar evidence applies to emotions, some of the processing for which can occur without conscious awareness (Phelps and LeDoux, 2005; LeDoux, 2008; Tamietto et al., 2009; Brown et al., 2019). Consistent with the hypothesis of multiple routes to action, only some of which involve conscious awareness, is the evidence that split-brain patients may not be aware of actions being performed by the ‘non-dominant’ hemisphere (Gazzaniga and LeDoux, 1978; Gazzaniga, 1988, 1995). Also consistent with multiple, including non-verbal, routes to action, patients with focal brain damage, for example to the prefrontal cortex, may emit actions, yet comment verbally that they should not be performing those actions (Rolls et al., 1994a; Hornak et al., 2003). In both these types of patient, confabulation may occur, in that a verbal account of why the action was performed may be given, and this may not be related at all to the environmental event that actually triggered the action.

Implicit (not phenomenally conscious) actions could involve control of behavior by brain systems that are old in evolutionary terms such as the basal ganglia. It is of interest that the basal ganglia (and cerebellum) do not have backprojection systems to most of the parts of the cerebral cortex from which they receive inputs (Rolls, 2016). In contrast, parts of the brain such as the hippocampus and amygdala, involved in functions such as episodic memory and emotion respectively, about which we can make (verbal) declarations (hence declarative memory; Squire, 1992) do have major backprojection systems to the high parts of the cerebral cortex from which they receive forward projections (Rolls, 2016). I suggest that evolutionarily newer parts of the brain, such as the language areas and parts of the prefrontal cortex, are involved in an alternative type of control of behavior, in which actions can be planned with the use of a (language) system that allows relatively arbitrary (syntactic) manipulation of semantic entities (symbols) (Rolls, 2016).

The general view that there are many routes to behavioral output is supported by the evidence that there are many input systems to the basal ganglia (from almost all areas of the cerebral cortex), and that neuronal activity in each part of the striatum reflects the activity in the overlying cortical area (Rolls, 2016). The evidence is consistent with the possibility that different cortical areas, each specialized for a different type of computation, have their outputs directed to the basal ganglia, which then select the strongest input, and map this into action (via outputs directed, for example, to the premotor cortex). The view is also supported by the evidence that the cingulate cortex is involved in actions performed for goals (Rolls, 2019a). Within this scheme, the language areas would offer one of many routes to action, but a route particularly suited to planning actions, because of the syntactic manipulation of semantic entities that may make long-term planning possible.

**FIGURE 1:**
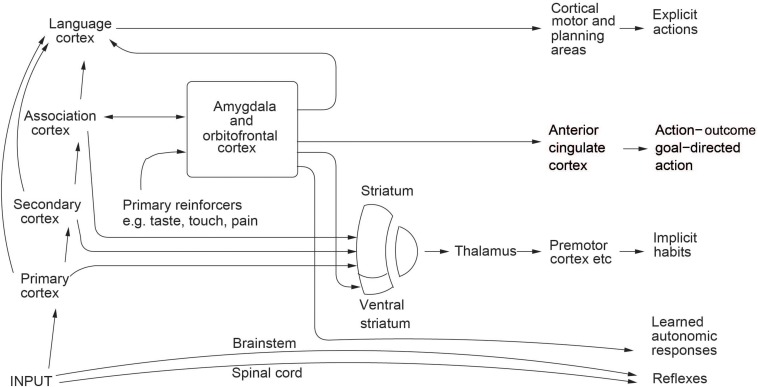
Multiple routes to the initiation of actions and other behavioral responses in response to rewarding and punishing stimuli. The inputs from different sensory systems to brain structures such as the orbitofrontal cortex and amygdala allow these brain structures to evaluate the reward- or punishment-related value of incoming stimuli, or of remembered stimuli. One type of route is via the language systems of the brain, which allow explicit (verbalizable) decisions involving multistep syntactic planning to be implemented. The other type of route may be implicit, and includes the anterior cingulate cortex for action–outcome, goal-dependent, learning; and the striatum and rest of the basal ganglia for stimulus–response habits. The basal ganglia may be involved in selecting only one system for output. Outputs for autonomic responses can also be produced using outputs from the orbitofrontal cortex and anterior cingulate cortex (some of which are routed via the anterior insular cortex) and amygdala.

It is accordingly possible that sometimes in normal humans when actions are initiated as a result of processing in a specialized brain region such as those involved in some types of rewarded behavior, the language system may subsequently elaborate a coherent account of why that action was performed (i.e., confabulate). This would be consistent with a general view of brain evolution in which, as areas of the neocortex evolve, they are laid on top of existing circuitry connecting inputs to outputs, and in which each level in this hierarchy of separate input–output pathways may control behavior according to the specialized function it can perform (Rolls, 2016) (see schematic in Figure 1). (It is of interest that mathematicians may get a hunch that something is correct, yet not be able to verbalize why. They may then resort to formal, more serial and language-like, theorems to prove the case, and these seem to require conscious processing. This is a further indication of a close association between linguistic processing, and consciousness. The linguistic processing need not, as in reading, involve an inner articulatory loop).

We may next examine some of the advantages and behavioral functions that language, present as the most recently added layer to the above system, would confer.

One major advantage would be the ability to plan actions through many potential stages and to evaluate the consequences of those actions without having to perform the actions. For this, the ability to form propositional statements, and to perform syntactic operations on the semantic representations of states in the world, would be important.

Also important in this system would be the ability to have second-order thoughts about the type of thought that I have just described (e.g., I think that she thinks that…, involving ‘theory of mind’), as this would allow much better modeling and prediction of others’ behavior, and therefore of planning, particularly planning when it involves others. [Second-order thoughts are thoughts about thoughts. Higher-order thoughts refer to second-order, third-order, etc., thoughts about thoughts… (A thought may be defined briefly as an intentional mental state, that is a mental state that is about something. Thoughts include beliefs, and are usually described as being propositional (Rosenthal, 2005). An example of a thought is “It is raining.” A more detailed definition is as follows. A thought may be defined as an occurrent mental state (or event) that is intentional – that is a mental state that is about something – and also propositional, so that it is evaluable as true or false. Thoughts include occurrent beliefs or judgments. Examples of thoughts are an occurrent belief that the earth moves around the sun; and that it never rains in southern California.)] This capability for higher-order thoughts would also enable reflection on past events, which would also be useful in planning. In contrast, non-linguistic behavior would be driven by learned reinforcement associations, learned rules etc., but not by flexible planning for many steps ahead involving a model of the world including others’ behavior. [The examples of behavior from non-humans that may reflect planning may reflect much more limited and inflexible planning. For example, the dance of the honey-bee to signal to other bees the location of food may be said to reflect planning, but the symbol manipulation is not arbitrary. There are likely to be interesting examples of non-human primate behavior that reflect the evolution of an arbitrary symbol-manipulation system that could be useful for flexible planning (Cheney and Seyfarth, 1990; Whiten and Byrne, 1997)].

It is important to state that the language ability referred to here is not necessarily human verbal language (though this would be an example). What it is suggested is important to planning is the syntactic manipulation of symbols, and it is this syntactic manipulation of symbols that is the sense in which language is defined and used here. The type of syntactic processing need not be at the natural language level (which implies a universal grammar), but could be at the level of mentalese (Fodor, 1994; Rolls, 2016).

I understand reasoning, and rationality, to involve syntactic manipulations of symbols in the way just described. Reasoning thus typically may involve multiple steps of ‘if… then’ conditional statements, all executed as a one-off or one-time process (see below), and is very different from associatively learned conditional rules typically learned over many trials, such as ‘if yellow, a left choice is associated with reward.’

### A Computational Hypothesis of Phenomenal Consciousness

Higher order thought (HOT) theory proposes that consciousness may *be* the state that arises in a system that can think about (or reflect on) its own (or other peoples’) thoughts, that is in a system capable of second- or higher-order thoughts ([citeskum]BR105,BR106,BR107,BR108,BR109,BR104[citeekum]Rosenthal, 1986, 1990, 1993, 2004, 2005, 2012; Dennett, 1991; Gennaro, 2004; [citeskum]BR75,BR76,BR77,BR78,BR79,BR80,BR82,BR85[citeekum]Rolls, 2004, 2007a, 2007b, 2008, 2010, 2011, 2012b, 2016; Lau and Rosenthal, 2011; Brown et al., 2019). On this account, a mental state is non-introspectively (i.e., non-reflectively) conscious if one has a roughly simultaneous thought that one is in that mental state. Following from this, introspective consciousness (or reflexive consciousness, or self consciousness) is the attentive, deliberately focused consciousness of one’s mental states. It is noted that not all of the higher-order thoughts need themselves be conscious (many mental states are not). However, according to the analysis, having a higher-order thought about a lower-order thought is necessary for the lower-order thought to be conscious (Rosenthal, 2005).

Rolls’ HOST theory of consciousness takes a more computational approach, and argues that there is evolutionary adaptive value in having a HOST computational mechanism that can correct first (or lower) order multistep syntactic plans, in which there is a credit assignment problem, as described below; and proposes that phenomenal consciousness is a state that is present when this computational module is engaged ([citeskum]BR75,BR76,BR77,BR78,BR79,BR80,BR82,BR85,BR86[citeekum]Rolls, 2004, 2007a, 2007b, 2008, 2010, 2011, 2012b, 2016, 2018). The symbols being processed by this HOST system need to be grounded in the world, as described below. This HOST theory of phenomenal consciousness is described in the rest of Section “A Higher Order Syntactic Thought (HOST) Theory of Phenomenal Consciousness.”

The brain systems that are required for consciousness and language are similar in at least some respects, I propose. In particular, a system that can have second- or higher-order thoughts about its own operation, including its planning and linguistic operation, must itself be a language processor, in that it must be able to bind correctly to the symbols and syntax in the first-order system. According to this explanation, the feeling of anything is the state that is present when a computational module is engaged that can perform linguistic and in particular syntactic processing that involves second- or higher-order thoughts about lower order syntactic thoughts.

It might be objected that this hypothesis captures some of the process aspects of consciousness, that is, what is useful in an information processing system, but does not capture the phenomenal aspect of consciousness. I agree that there is an element of an emergent property that is invoked at this step of the argument, when I say that it feels like something for a computational module with higher-order syntactic thoughts to be thinking about its own first- or lower-order syntactic thoughts. But the elaboration of this point is the following: if a human with second-order syntactic thoughts is thinking about her own first-order syntactic thoughts, surely it is very difficult for us to conceive that this would not feel like something? (Perhaps the higher-order thoughts in thinking about the first-order thoughts would need to have in doing this some sense of continuity or self, so that the first-order thoughts would be related to the same system that had thought of something else a few minutes ago. But even this continuity aspect may not be a requirement for consciousness. Humans with anterograde amnesia cannot remember what they felt a few minutes ago, yet their current state does feel like something).

As a point of clarification, I note that according to my theory, a language processing system [let alone a working memory system as proposed by LeDoux (2008)] is not sufficient for consciousness. What defines a conscious system according to this analysis is the ability to have higher-order syntactic thoughts, and a first order language processor (that might be perfectly competent at language) would not be conscious, in that it could not think about its own or others’ thoughts. One can perfectly well conceive of a system that obeyed the rules of language (which is the aim of some connectionist modeling), and implemented a first-order linguistic system, that would not be conscious. [Possible examples of language processing that might be performed non-consciously include computer programs implementing aspects of language, or ritualized human conversations, e.g., about the weather. These might require syntax and correctly grounded semantics, and yet be performed non-consciously. A more complex example, illustrating that syntax could be used, might be “If A does X, then B will probably do Y, and then C would be able to do Z.” A first order language system could process this statement. Moreover, the first order language system could apply the rule usefully in the world, provided that the symbols in the language system (A, B, X, Y etc.) are grounded (have meaning) in the world].

A second clarification is that the plan would have to be a unique string of steps, in much the same way as a sentence can be a unique and one-off (or one-time) string of words. The point here is that it is helpful to be able to think about particular one-off plans, and to correct them; and that this type of operation is very different from the slow learning of fixed rules by trial and error, or the application of fixed rules by a supervisory part of a computer program.

### Adaptive Value of the Computational Processing That Is Related to Phenomenal Consciousness

It is suggested that part of the evolutionary **adaptive significance** of this type of higher-order syntactic thought computational system is that it enables correction of errors made in first-order linguistic or in non-linguistic processing. Indeed, the ability to reflect on previous events is extremely important for learning from them, including setting up new long-term semantic structures. There is evidence that the hippocampus is a system for such ‘declarative’ recall of recent memories (Squire and Zola, 1996; Kesner and Rolls, 2015; Rolls, 2016). Its close relation to ‘conscious’ processing in humans (Squire has classified it as a declarative memory system) may be simply that it enables the recall of recent memories, which can then be reflected upon in conscious, higher-order, processing. Another part of the adaptive value of a higher-order thought system may be that by thinking about its own thoughts in a given situation, it may be able to understand better the thoughts of another individual in a similar situation, and therefore predict that individual’s behavior better (Humphrey, 1986).

In line with the argument on the adaptive value of higher-order thoughts and thus consciousness given above, that they are useful for correcting lower-order thoughts, I now suggest that correction using higher-order thoughts of lower-order thoughts would have adaptive value primarily if the lower-order thoughts are sufficiently complex to benefit from correction in this way. The nature of the complexity is specific – that it should involve syntactic manipulation of symbols, probably with several steps in the chain, and that the chain of steps should be a one-off (or in American usage, ‘one-time,’ meaning used once) set of steps, as in a sentence or in a particular plan used just once, rather than a set of well learned rules. The first or lower-order thoughts might involve a linked chain of ‘if… then’ statements that would be involved in planning, an example of which has been given above, and this type of cognitive processing is thought to be a primary basis for human skilled performance. It is partly because complex lower-order thoughts such as these that involve syntax and language would benefit from correction by higher-order thoughts that I suggest that there is a close link between this reflective consciousness and language.

The **computational hypothesis** is that by thinking about lower-order thoughts, the higher-order thoughts can discover what may be weak links in the chain of reasoning at the lower-order level, and having detected the weak link, might alter the plan, to see if this gives better success. In our example above, if it transpired that C could not do Z, how might the plan have failed? Instead of having to go through endless random changes to the plan to see if by trial and error some combination does happen to produce results, what I am suggesting is that by thinking about the previous plan, one might, for example, using knowledge of the situation and the probabilities that operate in it, guess that the step where the plan failed was that B did not in fact do Y. So by thinking about the plan (the first- or lower-order thought), one might correct the original plan in such a way that the weak link in that chain, that ‘B will probably do Y,’ is circumvented.

To draw a parallel with neural networks: there is a **credit assignment** problem in such multistep syntactic plans, in that if the whole plan fails, how does the system assign credit or blame to particular steps of the plan? [In multilayer neural networks, the credit assignment problem is that if errors are being specified at the output layer, the problem arises about how to propagate back the error to earlier, hidden, layers of the network to assign credit or blame to individual synaptic connections (Rumelhart et al., 1986; Rolls, 2016).] **My hypothesis is that this solution to the credit assignment problem for a one-off syntactic plan is the function of higher-order syntactic thoughts, and is why computational systems with higher-order syntactic thoughts evolved. The suggestion I then make is that if a system were doing this type of processing (thinking about its own thoughts), it would then be very plausible that it should feel like something to be doing this**. I even suggest to the reader that it is not plausible to suggest that it would not feel like anything to a system if it were doing this.

I emphasize that the plan would have to be a unique string of steps, in much the same way as a sentence can be a unique and one-off string of words. The point here is that it is helpful to be able to think about particular one-off plans, and to correct them; and that this type of operation is very different from the slow learning of fixed rules by trial and error, or the application of fixed rules by a supervisory part of a computer program.

### Symbol Grounding

A further point in the argument should be emphasized for clarity. The system that is having syntactic thoughts about its own syntactic thoughts (higher-order syntactic thoughts or HOSTs) would have to have its symbols grounded in the real world for it to feel like something to be having higher-order thoughts. The intention of this clarification is to exclude systems such as a computer running a program when there is in addition some sort of control or even overseeing program checking the operation of the first program. We would want to say that in such a situation it would feel like something to be running the higher level control program only if the first-order program was symbolically performing operations on the world and receiving input about the results of those operations, and if the higher-order system understood what the first order system was trying to do in the world.

The symbols (or symbolic representations) are symbols in the sense that they can take part in syntactic processing. The symbolic representations are grounded in the world in that they refer to events in the world. The symbolic representations must have a great deal of information about what is referred to in the world, including the quality and intensity of sensory events, emotional states, etc. The need for this is that the reasoning in the symbolic system must be about stimuli, events, and states, and remembered stimuli, events and states, and for the reasoning to be correct, all the information that can affect the reasoning must be represented in the symbolic system, including for example just how light or strong the touch was, etc. Indeed, it is pointed out (Rolls, 2014a) that it is no accident that the shape of the multidimensional phenomenal (sensory etc.) space does map so clearly onto the space defined by neuronal activity in sensory systems, for if this were not the case, reasoning about the state of affairs in the world would not map onto the world, and would not be useful. Good examples of this close correspondence are found in the taste system, in which subjective space maps simply onto the multidimensional space represented by neuronal firing in primate cortical taste areas (Kadohisa et al., 2005). In particular, if a two-dimensional space reflecting the distances between the representations of different tastes provided by macaque neurons in the cortical taste areas is constructed, then the distances between the subjective ratings by humans of different tastes is very similar (Kadohisa et al., 2005; Rolls, 2014b). Similarly, the changes in human subjective ratings of the pleasantness of the taste, smell and sight of food parallel very closely the responses of neurons in the macaque orbitofrontal cortex (Rolls, 2014a).

The representations in the first-order linguistic processor that the HOSTs process include beliefs (for example “Food is available,” or at least representations of this), and the HOST system would then have available to it the concept of a thought (so that it could represent “I believe [or there is a belief] that food is available”). However, as argued by Rolls (2014a), representations of sensory processes and emotional states must be processed by the first-order linguistic system, and HOSTs may be about these representations of sensory processes and emotional states capable of taking part in the syntactic operations of the first-order linguistic processor. Such sensory and emotional information may reach the first-order linguistic system from many parts of the brain, including those such as the orbitofrontal cortex and amygdala implicated in emotional states. When the sensory information is about the identity of the taste, the inputs to the first-order linguistic system must come from the primary taste cortex, in that the identity and intensity of taste, independently of its pleasantness (in that the representation is independent of hunger) must come from the primary taste cortex. In contrast, when the information that reaches the first-order linguistic system is about the pleasantness of taste, it must come from the secondary taste (orbitofrontal) cortex, in that there the representation of taste depends on hunger and is linearly related to pleasantness (Grabenhorst and Rolls, 2008; Rolls and Grabenhorst, 2008; Rolls, 2014a).

The main answer that I propose now to the issue of symbol grounding is as follows, with more details available (Rolls, 2016). The gene-specified rewards or goals for action that are the bases for emotional and motivational states play the role for these states of grounding them in the world. The organism has to be built to want food rewards, to avoid pain, etc. I propose that this grounding for gene-specified emotional and motivational states provides the basis for the symbol grounding in the symbolic system, in that what the symbolic system computes, in a sense its goals, must be close to what the gene-specified emotional and motivational systems are grounded in, as otherwise the symbolic reasoning system and the gene goal-based emotional system would be inconsistent, and the reasoning system would not have adaptive value in evolution. To put this another way, unless the symbolic syntactic reasoning system had the belief that the gene-specified goals of the emotional system were among the goals of the reasoning system, then the two systems together could not produce consistent actions in general, and that would be unadaptive in the evolutionary sense. That leaves it open then in evolution for the symbolic system to add reasoned goals of its own, which might be for the advantage of the individual, but would still be in the same design framework of the emotion- and motivation-related goals specified by genes (Rolls, 2014a, 2018).

### Qualia: Phenomenal Aspects of Consciousness

This analysis does not yet give an account for sensory qualia (raw sensory feels, for example why ‘red’ feels red), for emotional qualia (e.g., why a rewarding touch produces an emotional feeling of pleasure), or for motivational qualia (e.g., why food deprivation makes us feel hungry). The view I suggest on such **qualia** is as follows.

Information processing in and from our sensory systems (e.g., the sight of the color red) may be relevant to planning actions using language and the conscious processing thereby implied. Given that these inputs must be represented in the system that plans, we may ask whether it is more likely that we would be conscious of them or that we would not. I suggest that it would be a very special-purpose system that would allow such sensory inputs, and emotional and motivational states, to be part of (linguistically based) planning, and yet remain unconscious (given that the processing being performed by this system is inherently conscious, as suggested above). It seems to be much more parsimonious to hold that we would be conscious of such sensory, emotional, and motivational qualia because they would be being used (or are available to be used) in this type of (linguistically based) higher-order thought processing system, and this is what I propose.

The explanation of emotional and motivational subjective feelings or qualia that this discussion has led toward is thus that they should be felt as conscious because they enter into a specialized linguistic symbol-manipulation system, which is part of a higher-order thought system that is capable of reflecting on and correcting its lower-order thoughts involved for example in the flexible planning of actions. It would require a very special machine to enable this higher-order linguistically-based thought processing, which is conscious by its nature, to occur without the sensory, emotional and motivational states (which must be taken into account by the higher-order thought system) becoming felt qualia. The sensory, emotional, and motivational qualia are thus accounted for by the evolution of a linguistic (i.e., syntactic) system that can reflect on and correct its own lower-order processes, and thus has adaptive value. Reasons why the ventral visual system is more closely related to explicit (conscious) than implicit processing include the fact that representations of objects and individuals need to enter the planning, hence conscious, system, and are considered in more detail elsewhere (Rolls, 2003, 2016).

This account implies that it may be especially animals with a higher-order belief and thought system and with linguistic (i.e., syntactic, not necessarily verbal) symbol manipulation that have qualia. It may be that much non-human animal behavior, provided that it does not require flexible linguistic planning and correction by reflection, could take place according to reinforcement-guidance (using, e.g., stimulus-reinforcer association learning in the amygdala and orbitofrontal cortex (Rolls, 2014a, 2019b), and rule-following [implemented, e.g., using habit or stimulus-response learning in the basal ganglia]). Such behaviors might appear very similar to human behavior performed in similar circumstances, but need not imply qualia. It would be primarily by virtue of a system for reflecting on flexible, linguistic, planning behavior that humans (and animals close to humans, with demonstrable syntactic manipulation of symbols, and the ability to think about these linguistic processes) would be different from other animals, and would have evolved qualia.

It is of interest to comment on how the evolution of a system for flexible planning might affect emotions (Rolls, 2018). Consider grief which may occur when a reward is terminated and no immediate action is possible. It may be adaptive by leading to a cessation of the formerly rewarded behavior and thus facilitating the possible identification of other positive reinforcers in the environment. In humans, grief may be particularly potent because it becomes represented in a system which can plan ahead, and understand the enduring implications of the loss. Thus depression in humans may be much more severe than in animals without a reasoning system, because the explicit, reasoning, system can see how bad the non-reward or punisher really is, can foresee the consequences for the future using reasoning, and using re-entrant processing between the explicit and implicit systems may produce positive feedback as a result of rumination (Rolls, 2018). In this situation, thinking about or verbally discussing emotional states may help, because this can lead toward the identification of new or alternative reinforcers, and of the realization that for example negative consequences may not be as bad as feared.

### How Might HOST Processing Be Implemented in the Brain?

A key requirement of the implementation of HOST processing is that it needs to allow manipulation of items in some type of short-term memory store, so that for example several steps in a plan can be evaluated, and rearranged, or corrected. Now much of the neurophysiology of short-term memory has been on how individual items can be stored and later recalled (Goldman-Rakic, 1996; Rao et al., 1997; Miller and Cohen, 2001; Miller, 2013; Lundqvist et al., 2018), utilizing continuing firing in attractor networks for both single items and multiple items (Deco and Rolls, 2003, 2005a; Deco et al., 2010; Rolls and Deco, 2010; Martinez-Garcia et al., 2011; Rolls et al., 2013; Rolls, 2016). A key brain area for such short term memory systems is the prefrontal cortex (Passingham and Wise, 2012). An ordered temporal sequence of items can also be encoded in some brain systems such as the hippocampus (Eichenbaum, 2014, 2017; Howard and Eichenbaum, 2015), by taking advantage of neurophysiological processes such as synaptic or neuronal temporal adaptation to provide a basis for a temporal order mechanism (Deco and Rolls, 2005c; Rolls and Mills, 2019).

However, psychologists have long distinguished between short-term memory and working memory, with the latter having the property that items in the working memory can be manipulated, for example rearranged into a different order (Baddeley, 2007, 2012; Baddeley et al., 2019). Given that I see the HOST system as having usefulness for correcting and re-arranging, in fact manipulating, items that might be multiple steps of a plan, working memory may be a component required for HOST computations. The prefrontal cortex is implicated in working memory (Passingham and Wise, 2012), and that points toward the prefrontal cortex as a candidate region for at least some role in HOST computations. Moreover, investigations of which brain regions may become involved when processing becomes sufficiently global when phenomenal consciousness is present include working memory-related regions of the prefrontal cortex (Dehaene, 2014).

Now the manipulation of items (which are likely to be symbols) in a HOST system requires at least some syntax, for different symbols may appear in different steps of a multiple step plan, and some symbols may be in several steps. Now if we consider which parts of the prefrontal cortex may be related to symbol manipulation in humans, the left inferior frontal gyrus, Broca’s area, BA45 and BA44 is the most obvious region. Indeed, it may be involved in syntactical processing in humans, with perhaps the role of ordering words to produce a stream of words with the items in the stream ordered grammatically correctly (Amunts and Zilles, 2012). Moreover, the human left inferior frontal gyrus has major connectivity with the temporal lobe areas implicated in semantic memory (Kelly et al., 2010; Petrides, 2014; Du et al., 2020), and it may be from these temporal lobe areas that the semantics is obtained that may be woven into a syntactic stream by the left inferior frontal gyrus. The connectivity of the human left inferior frontal gyrus with the angular and supramarginal gyri (Petrides, 2014; Du et al., 2020) also provides evidence that the human inferior frontal gyrus plays a key role in linguistic processing, which at least requires syntax.

How syntactical operations, necessary for HOST processing, but not necessarily at the level of human linguistic processing, are implemented in the brain remains a major challenge for neuroscience (Rolls, 2016). One proposal, consistent with much of what we know about connected cortical attractor networks, is that syntax is implemented by a trajectory through a state space in one direction because of stronger forward than backward connections between cortical modules, with each module specialized for a different syntactical role, such as subject or verb or object (Rolls and Deco, 2015; Rolls, 2016). Each such cortical module is a small cortical region approximately 2–3 mm in diameter in which there is a reasonably high probability (0.1) of connections between the neurons, so that an attractor network can be implemented. Each such neocortical module has in the order of 10,000 connections onto every pyramidal neuron devoted to these local recurrent collateral connections, and an attractor network of this type has a capacity of in the order of 10,000 items (e.g., nouns, or verbs) (given somewhat sparse representations), as has been proved with the tools of theoretical physics (Amit, 1989; Treves, 1991; Treves and Rolls, 1991) and confirmed by simulation (Rolls et al., 1997; Rolls, 2012a, 2016). This is of considerable interest, for any one such small cortical module has the capacity to store, and produce when cued, in the order of 10,000 items, which is in the order of the number of words in a typical vocabulary (Rolls and Deco, 2015). This approach has been developed as far as a working computational model using biologically plausible integrate-and-fire neuronal networks of how syntax might be implemented in the brain (Rolls and Deco, 2015). However, a limitation is that this approach has not yet been developed beyond simple sentences with a subject, verb, and object, using temporal order as a way in which the syntax is encoded by the sender, and decoded by the receiver (Rolls and Deco, 2015).

This is thus one possible way to think about how the syntax required for HOST processing might be implemented in the brain; but research on computational mechanisms for syntax in the brain is at an early stage; and the left inferior frontal gyrus might be one of a number of connected cortical areas that play a role in the syntactic processing required for HOSTs in humans (Rolls, 2021).

### Conscious Free Will

Our brains operate non-deterministically, and in that sense we might be said to be free (Rolls, 2010, 2016; Rolls and Deco, 2010). The non-deterministic computations performed by the brain are due to the almost random spike times for a given mean firing rate: the spike times have an almost Poisson distribution, apart from the refractory period, during the normal operation of the cerebral cortex during waking (Rolls, 2016). This stochasticity arises from the fact that the membrane potential is held close to the threshold of firing, so that if some extra synaptic input arrives at a neuron, it can fire spikes very soon, without having to wait for the membrane potential with its 20 ms time constant to be driven up toward the threshold of firing (Rolls, 2016); and there is always a little noise in the system, arising for example from ion channel noise (Faisal et al., 2008). This stochasticity results in decision-making being probabilistic if the odds are close to even. This stochasticity also results in jumps in memory systems in the brain, from one representation to another, allowing a certain stochasticity in thought processes (Rolls and Deco, 2010; Rolls, 2016). Although the noise is random, the jumps are not random, but the probability of their direction in the high dimensional space of stored associative memories, for example semantic memories, depends on the strength of the connections between the nodes. In a semantic memory, a jump might be from one idea to a somewhat related idea. I have argued that this is a key feature of human creativity, and the fact that the jumps are not in random directions helps us to discover new ideas around a particular theme, instead of just filling our minds with random sets of ideas. After a jump in such an associative memory, it is up to the reasoning (rational) system to evaluate whether the new idea is useful, or whether to abandon it and keep thinking. All of this has been worked out in detail with the concepts of theoretical physics and with integrate-and-fire neuronal network simulations (Deco et al., 2009; Rolls, 2010, 2016; Rolls and Deco, 2010). Creativity has even been linked to the variability of functional connectivity, which is produced by these mechanisms (Sun et al., 2019). The point in relation to free will is that, unlike most computers, we do not perform fully deterministic computations, and in that sense have some freedom of thought, and of the decisions that are taken, by this combination of stochasticity and rational evaluation of the results of the stochasticity.

In Section “**Multiple Routes to Action**” on multiple routes to action, it was argued that one type of route is implicit, that is unconscious, and accounts for much of our behavior. All of the evidence from subliminal processing (i.e., without consciousness), and inattentional blindness, attests to this (Dehaene, 2014; Dehaene et al., 2014), as does work on split-brain patients (Gazzaniga and LeDoux, 1978). The explicit, reasoning, system may confabulate explanations about why such implicit actions were performed. But when the reasoning (explicit, rational) system, using syntactic manipulation of symbols, is being used, then the reasoning for the decision that is given may be a true reflection of the computations being performed. Further, such decisions can be in the interests of the individual person, or could be perfectly altruistic, whereas decisions taken by the implicit system will generally be in the interests of the genotype, with the arguments elaborated elsewhere (Rolls, 2014a, 2016, 2018). The point I make here though is that if we wish to use the term free will, then it could be applied to the rational system, in which decisions can be conscious, as described here.

## Comparison With Other Theories of Consciousness

### Global Workspace Theories of Consciousness

My HOST theory of phenomenal consciousness described here hypothesizes that the information must be being processed in a computational system capable of implementing HOSTs for the information to be phenomenally conscious, and is in this sense more specific than global workspace hypotheses (Baars, 1988; Cole et al., 2014; Dehaene et al., 2014, 2017; Carruthers, 2019). I suggest that a workspace could be sufficiently global for the complex processing involved in for example driving a car, and yet the processing might be performed unconsciously, unless there was activity about the driving in the HOST (supervisory, monitory, correcting) system. A key weakness of global workspace theories of consciousness is that there is no precise way of prescribing just what level of globalness is needed for what is considered to be the all-or-none kind of phenomenal consciousness to be present. HOST theory in contrast holds that there is a particular kind of computation that is needed for phenomenal consciousness to be present, and so can make precise predictions about the brain mechanisms related to consciousness. However, research taking the approach of a global workspace is very helpful in providing evidence on which brain regions become particularly involved when phenomenal consciousness is present (Dehaene and Naccache, 2001; Dehaene, 2014; Dehaene et al., 2014, 2017).

A number of issues have been raised above about Carruthers’s (2019) version of global workspace theory. One is that conceptual as well as non-conceptual content can be present on phenomenal conscious. Another is that, given the levels of explanation points made in Section “Attention and Phenomenal Consciousness,” I do not hold the view that phenomenal consciousness can be reduced to physical properties, although as was made clear, the physical processes at a lower level than that of phenomenal consciousness must take place for phenomenal consciousness to arise as an emergent property. Carruthers also does not specify any utility for phenomenal consciousness, whereas I have argued in Section “Adaptive Value of the Computational Processing That Is Related to Phenomenal Consciousness” that the HOST-related computation that is associated with phenomenal consciousness is computationally very useful, as it is part of the way in which credit assignment problems for errors in multistep syntactic processing can be corrected.

### Higher Order Thought (HOT) Theories

Higher order thought theories of phenomenal consciousness hold that a HOT is needed to make a first order thought conscious (Rosenthal, 1990, 2004, 2005, 2012; Carruthers, 2000; Gennaro, 2004; Lau and Rosenthal, 2011). LeDoux has moved away from his former working memory approach to consciousness (LeDoux, 2008) toward the HOT theory of consciousness with colleagues (Brown et al., 2019). Some ways in which my HOST theory is different from other HOT theories is that it provides a computational account of the evolutionary, adaptive, value of a higher-order syntactic thought system. I propose that the HOST system has the capability to solve the credit assignment problem that arises in a multistep syntactic plan, and links this type of computation to phenomenal consciousness, and therefore emphasizes a role for syntactic processing in consciousness. The type of syntactic processing implemented in the HOST system need not be at the natural language level (which implies a universal grammar), but could be at the level of mentalese, or simpler, as it involves primarily the syntactic manipulation of symbols and not necessarily the production of speech (Fodor, 1994; Rolls, 2016).

My theory holds that it is higher-order syntactic thoughts, HOSTs ([citeskum]BR72,BR73,BR75,BR76,BR77,BR78,BR82,BR85,BR86[citeekum]Rolls, 1997a, b, 2004, 2007a, 2007b, 2008, 2012b, 2016, 2018) that are closely associated with consciousness, and may differ from Rosenthal’s higher-order thought (HOT) theory (Rosenthal, 1990, 2004, 2005, 2012; Lau and Rosenthal, 2011) in the emphasis in my theory of the syntactic manipulation of symbols, which is useful for example in thinking through and comparing plans, and in credit assignment. The computational module suggested includes a type of working memory with these capabilities.

The reason that syntax is emphasized is that it is as a result of having a multistep, flexible, ‘one-off’, reasoning procedure that errors can be corrected by using ‘thoughts about thoughts’. This enables correction of errors that cannot be easily corrected by reward or punishment received at the end of the reasoning, due to the credit assignment problem. That is, there is a need for some type of supervisory and monitoring process, to detect where errors in the multi-step reasoning have occurred. According to HOST theory, phenomenal consciousness about information arises when the HOST computational brain system becomes engaged in processing that information. The nature of the representation of information in the brain is described in *Cerebral Cortex; Principles of Operation* (Rolls, 2016).

This suggestion on the adaptive value in evolution of such a higher-order linguistic thought process for multistep planning ahead, and correcting such plans, may also be different from earlier work. The computational point is that **credit assignment** when reward or punishment is received is straightforward in a one-layer network (in which the reinforcement can be used directly to correct nodes in error, or responses) [see Appendix 2 of Rolls (2016)], but is very difficult in a multistep linguistic process executed once. Very complex mappings in a multilayer network can be learned but only with hundreds of training trials, and with an explicit teaching signal (LeCun et al., 2015; Rolls, 2016). But once these complex mappings are learned, their success or failure in a new situation on a given trial cannot be evaluated and corrected by the network. Indeed, the complex mappings achieved by such networks (e.g., networks trained by backpropagation of errors or by reinforcement learning) mean that after training they operate according to fixed rules, and are often quite impenetrable, inflexible, and difficult to correct quickly (Rumelhart et al., 1986; Rolls, 2016; Plebe and Grasso, 2019). In contrast, to correct a multistep, single occasion, syntactically based plan, recall of the steps just made in the reasoning or planning, and perhaps of related episodic material, needs to occur, so that the step in the chain of reasoning that is most likely to be in error can be identified. This may be part of the reason why there is a close relationship between declarative memory systems, which can explicitly recall memories, so that the steps in a plan can for example be corrected, and consciousness.

Some computer programs may have supervisory processes. Should these count as higher-order linguistic thought processes? I do not think that they should, to the extent that they operate with fixed rules to correct the operation of a system that does not involve one-off syntactic thoughts about symbols grounded semantically in the external world. On the other hand, if it was possible to implement on a computer such a high-order syntactic thought-supervisory process to correct first-order one-off syntactic thoughts with symbols grounded in the real world, then prima facie I would argue that this process would be conscious. If it were possible in a thought experiment to reproduce the neural connectivity and computations performed by a human brain on a computer, then prima facie it would also have the attributes of consciousness. This is a functionalist position. Such a computer might continue to have those attributes for as long as power was applied to the system. Another possible difference from earlier theories is that raw sensory feels are suggested to arise when sensory information is being processed by this computational system that can think about its own thoughts. Raw sensory feels, and subjective states associated with emotional and motivational states, may not necessarily arise first in evolution.

A property often attributed to consciousness is that it is *unitary*. My HOST theory accounts for this by the limited syntactic capability of neuronal networks in the brain, which render it difficult to implement more than a few syntactic bindings of symbols simultaneously, and where the items being processed occur in a single serial processing stream (Rolls and Deco, 2015; Rolls, 2016). Such a stream might involve a serial trajectory through a linked set of attractor network nodes each of which is specialized for a different component of syntax (Rolls and Deco, 2015; Rolls, 2016). This limitation makes it difficult to run several ‘streams of consciousness’ simultaneously. In addition, given that a linguistic system can control behavioral output, several parallel streams might produce maladaptive behavior (apparent as, e.g., indecision, or attempting to perform incompatible responses simultaneously), and might be selected against. The close relationship between, and the limited capacity of, both the stream of consciousness, and a type of auditory-verbal short-term working memory, may be that both implement the capacity for syntax in neural networks. The difficulty of implementing syntactic binding in neuronal networks (Rolls and Deco, 2015; Rolls, 2016) may well place limitations on consciousness that lead to some of its properties, such as its unitary nature.

The HOST theory holds that consciousness arises by virtue of a computational system that can think syntactically about its own syntactic thoughts. The advantages for a system of being able to do this have been described, and this has been suggested as the reason why consciousness evolved. The evidence that consciousness arises by virtue of having a computational system that can perform higher-order syntactic processing is however, and I think may remain, circumstantial. (Why does it feel like something when we are performing a certain type of information processing? A plausibility argument may help: can one think of oneself thinking about, manipulating, and correcting one’s own multistep plans and thoughts that are grounded in the world without it feeling like something when such a computation is taking place?) The evidence, summarized above, includes the points that we think of ourselves as conscious when, for example, we recall earlier events, compare them with current events, and plan many steps ahead. Evidence also comes from neurological cases, from, for example, split-brain patients (who may confabulate conscious stories about what is happening in their other, non-language, hemisphere) (Gazzaniga and LeDoux, 1978); and from cases such as frontal lobe patients who can tell one consciously what they should be doing, but nevertheless may be doing the opposite (Rolls et al., 1994a; Rolls, 2014a). (The force of this type of case is that much of our behavior may normally be produced by routes about which we cannot verbalize, and about which we are not conscious).

This raises discussion of the *causal role of consciousness*. Does consciousness cause our behavior? The view that I currently hold is that the computation and information processing that is related to consciousness (activity in a linguistic system capable of higher-order thoughts, and used for planning and correcting the operation of lower-order linguistic systems) can play a causal role in producing our behavior (see Figure 1). The framework is what is described in the ‘levels of explanation’ account of the relation between mental events and brain events in Section “Levels of Explanation in Neuroscience, and the Implication That Phenomena at One Level, Such as Phenomenal Consciousness, Cannot be Reduced to What Co-occurs at a Lower Level, Such as Physical Processes.”

### Monitoring, Confidence, and Consciousness

**FIGURE 2:**
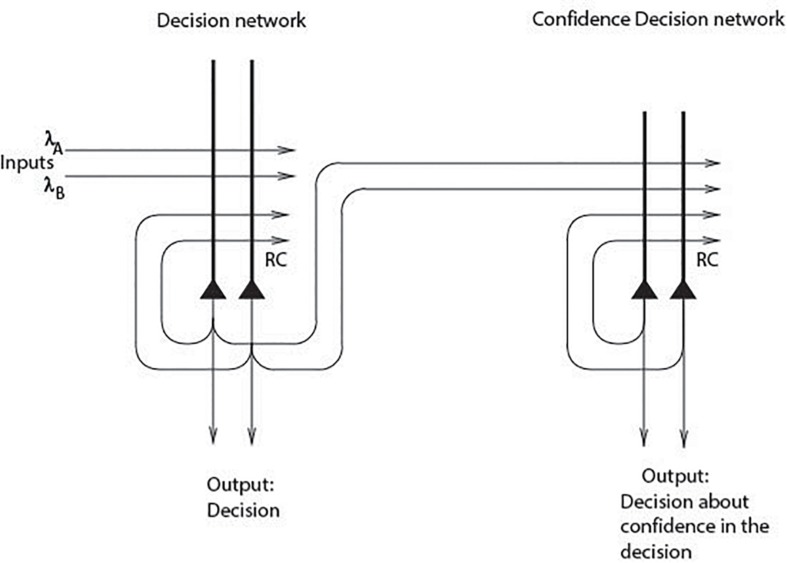
Network architecture for decisions about confidence estimates. The first network is a decision-making network, and its outputs are sent to a second network that makes decisions based on the firing rates from the first network, which reflect the decision confidence. In the first network, high firing of neuronal population (or pool) DA represents decision A, and high firing of population DB represents decision B. Pools DA and DB receive a stimulus-related input (respectively *λ*_A_ and *λ*_B_), the evidence for each of the decisions, and these bias the attractor networks, which have internal positive feedback produced by the recurrent excitatory connections (RC). Pools DA and DB compete through inhibitory interneurons. The neurons are integrate-and-fire spiking neurons with random spiking times (for a given mean firing rate) which introduce noise into the network and influence the decision-making, making it probabilistic. The neurons in the winning population of the first network have a higher firing rate for confident decisions in which the difference between the decision variables is large (Rolls et al., 2010a; Rolls, 2016). The second network is a confidence decision attractor network, and receives inputs from the first network. The confidence decision network has two selective pools of neurons, one of which (C) responds to represent confidence in the decision, and the other of which responds when there is little or a lack of confidence in the decision (LC). The C neurons receive the outputs from the selective pools of the (first) decision-making network, and the LC neurons receive *λ*_Reference_ which is from the same source but saturates at 40 spikes/s, a rate that is close to the rates averaged across correct and error trials of the sum of the firing in the selective pools in the (first) decision-making network. The second attractor network allows decisions to be made about whether to change the decision made by the first network, and for example abort the trial or strategy. The second network, the confidence decision network, is in effect monitoring the decisions taken by the first network, and can cause a change of strategy or behavior if the assessment of the decision taken by the first network does not seem a confident decision. From Insabato et al. (2010).

Attractor networks in the cerebral cortex with positive feedback implemented by excitatory recurrent collateral connections between the neurons can implement decision-making (Wang, 2002, 2008; Deco and Rolls, 2006; Rolls and Deco, 2010; Rolls, 2016). If the external evidence for the decision is consistent with the decision taken (which has been influenced by the noisy neuronal firing times), then the firing rates in the winning attractor are supported by the external evidence, and become especially high (Rolls and Deco, 2010; Rolls et al., 2010a, b; Rolls, 2016). If we now add a second attractor network to receive the firing rates from the first decision-making network, the second attractor network can take a decision based on the confidence expressed in the firing rates of the neurons in the first network (Insabato et al., 2010) (Figure 2). This shows that a type of monitoring can be performed in a simple system.

Now this is the type of description of, and language used, to describe ‘monitoring’ functions possibly related to consciousness (Block, 1995; Lycan, 1997; Hampton, 2001). However, we can account for what seem like complex cognitive phenomena with a simple system of two attractor decision-making networks (Insabato et al., 2010; Rolls, 2016). My argument is that some types of ‘self-monitoring’ are computationally simple, for example in decisions made based on confidence in a first decision, and may have little to do with consciousness; whereas HOST processes are very different in terms of the type of syntactic computation required, and may be more closely related to consciousness (Rolls, 2016).

If this type of computational system that can perform syntactic manipulations on symbols in a special type of working memory is related to phenomenal consciousness in humans, where does that leave the question about phenomenal consciousness in other animals (Dawkins, 1993, 2017; Carruthers, 2019), and in computers? First, I make it clear that our current understanding of phenomenal consciousness is very incomplete, and current theories should not be thought of as having implications. Second, I note that the usual criterion for phenomenal consciousness being present is a verbal report that it is (or a response made after verbal description that the response is to be made when the participant is phenomenally conscious). It is difficult to apply that criterion to non-human animals. In those circumstances, how might we investigate whether at least some animals have something like phenomenal consciousness? The clearest way I suggest would be to better understand exactly what neural computation is being performed in humans when they report being phenomenally conscious, and how that computation is implemented in the brain. Then if something similar is found in some animals, an inference would be that the computation has the same properties, including what it feels like. Indeed, as a functionalist, I believe that if the same computation could be implemented in a computer as that which is related to phenomenal consciousness in humans, then the computer would report feeling phenomenally conscious.

## Conclusion

Problems have been raised with the global workspace hypotheses of consciousness, for example about exactly how global the workspace needs to be for consciousness to suddenly be present, and that the hypothesis deals mainly with access consciousness (Dehaene, 2014). Problems have also been raised with a version (Carruthers, 2019) that excludes conceptual (categorical or discrete) representations, and in which phenomenal consciousness can be reduced to physical processes. Instead, I advocate a levels of explanation approach to the relation between the brain and the mind.

A different theory of phenomenal consciousness is described, in which there is a particular computational system involved in which HOSTs are used to perform credit assignment on first order thoughts of multiple step plans, to correct them by manipulating symbols in a syntactic type of working memory. This provides a good reason for the evolution of this kind of computational module, with which, it is proposed, phenomenal consciousness is associated. Some advantages of this HOST approach to phenomenal consciousness are then described with reference not only to the global workspace approach, but also to HOT theories. It is hypothesized that the HOST system which requires the ability to manipulate first order symbols in working memory might utilize parts of the prefrontal cortex implicated in working memory, and especially the left inferior frontal gyrus, which is involved in language and probably syntactical processing.

It appears that those involved in current global workspace theories may not be so distant from this perspective. At least Dehaene (2014) states that reasoning may involve consciousness: “rationally thinking about a problem.” That is starting to become close to HOST theory, in that at least Dehaene has in mind something to do with serial thinking of the type that might be implemented by a system capable of syntax, which might be a set of first order thoughts attempting to solve a problem. So first order thoughts of this syntactic type, supplemented I suggest by HOSTs useful to correct the first order thoughts, may be an area in which there may be convergence in future when thinking about phenomenal consciousness.

## Author Contributions

The author confirms being the sole contributor of this work and has approved it for publication.

## Conflict of Interest

The authors declare that the research was conducted in the absence of any commercial or financial relationships that could be construed as a potential conflict of interest.
